# Is guideline-adherent prescribing associated with quality of life in patients with type 2 diabetes?

**DOI:** 10.1371/journal.pone.0202319

**Published:** 2018-08-16

**Authors:** Kirsten P. J. Smits, Grigory Sidorenkov, Nanne Kleefstra, Steven H. Hendriks, Margriet Bouma, Marianne Meulepas, Gerjan Navis, Henk J. G. Bilo, Petra Denig

**Affiliations:** 1 Department of Clinical Pharmacy and Pharmacology, University Medical Center Groningen, Groningen, the Netherlands; 2 Langerhans Medical Research Group, Zwolle, the Netherlands; 3 Department of Internal Medicine, University Medical Center Groningen, Groningen, the Netherlands; 4 Diabetes Centre, Isala Clinics, Zwolle, the Netherlands; 5 Dutch College of General Practitioners (NHG), Utrecht, the Netherlands; 6 Dutch Institute for Rational Use of Medicine (IVM), Utrecht, the Netherlands; 7 Department of Nephrology, University Medical Center Groningen, Groningen, the Netherlands; Universidad Miguel Hernandez de Elche, SPAIN

## Abstract

**Background:**

Guideline-adherent prescribing for treatment of multiple risk factors in type 2 diabetes (T2D) patients is expected to improve clinical outcomes. However, the relationship to Health-Related Quality of Life (HRQoL) is not straightforward since guideline-adherent prescribing can increase medication burden.

**Objectives:**

To test whether guideline-adherent prescribing and disease-specific medication burden are associated with HRQoL in patients with T2D.

**Methods:**

Cross-sectional study including 1,044 T2D patients from the e-VitaDM/ZODIAC study in 2012 in the Netherlands. Data from the diabetes visit, such as laboratory and physical examinations and prescribed medication, and from two HRQoL questionnaires, the EuroQol 5 Dimensions 3 Levels (EQ5D-3L) and the World Health Organization Well-Being Index (WHO-5) were collected. Twenty indicators assessing prescribing of recommended glucose lowering drugs, statins, antihypertensives and renin-angiotensin-aldosterone system (RAAS)-inhibitors and potentially inappropriate drugs from a validated diabetes indicator set were included. Disease-specific medication burden was assessed using a modified version of the Medication Regimen Complexity Index (MRCI). Associations were tested with regression models, adjusting for age, gender, diabetes duration, comorbidity, body mass index and smoking.

**Results:**

The mean MRCI was 7.1, the median EQ5D-3L-score was 0.86 and the mean WHO-5 score was 72. Seven indicators included too few patients and were excluded from the analysis. The remaining thirteen indicators focusing on recommended start, intensification, current and preferred use of glucose lowering drugs, statins, antihypertensives, RAAS inhibitors, and on inappropriate prescribing of glibenclamide and dual RAAS blockade were not significantly associated with HRQoL. Finally, also the MRCI was not associated with HRQoL.

**Conclusions:**

We found no evidence for associations between guideline-adherent prescribing or disease-specific medication burden and HRQoL in T2D patients. This gives no rise to refrain from prescribing intensive treatment in T2D patients as recommended, but the interpretation of these results is limited by the cross-sectional study design and the selection of patients included in some indicators.

## Introduction

Clinical guidelines for managing patients with type 2 diabetes (T2D) recommend pharmacotherapy to reduce levels of risk factors such as glycated hemoglobin (HbA_1c_), blood pressure, low-density lipoprotein (LDL)-cholesterol and albuminuria [1, 2]. These recommendations are based on clinical trials assessing the efficacy and safety of these treatments. Patients receiving treatment according to these recommendations show improved intermediate [3] and hard clinical outcomes [4]. It is expected that improved clinical outcomes have a positive effect on health-related quality of life (HRQoL) in patients with T2D [5]. However, following treatment recommendations may also have a negative effect on HRQoL by increasing medication burden and inducing an increased risk for adverse drug events [6]. Medication burden proved to be negatively associated with physical and general HRQoL in various patient populations [7–9]. In addition, prescribing more medication may increase the risk of unsafe or inappropriate prescribing. Previously it was found that the use of inappropriate drugs is associated with reduced general and mental HRQoL in elderly patients [10]. Also, adverse drug events resulting from inappropriate drugs use can negatively influence HRQoL [8, 11].

Several studies have assessed the association between glucose lowering drugs and HRQoL. These studies found that prescribing of insulin may be associated with lower general but not mental HRQoL [12, 13] and prescribing of various oral glucose lowering drugs is not associated with differences in HRQoL [13, 14]. Furthermore, one study found that intensive multitherapy for glycemic, blood pressure and cholesterol control was associated with better general HRQoL compared with usual care [15]. On the other hand, another study found that an increase in the number of cardioprotective agents including glucose-, blood pressure- and cholesterol lowering drugs, did not change HRQoL in T2D patients [16]. Data on the effect of prescribing drug treatment other than glucose lowering drugs or potentially inappropriate drugs in T2D patients on HRQoL is unavailable.

Therefore, the primary aim of this study in T2D patients was to assess the relationship of (I) guideline-adherent prescribing of glucose lowering drugs, statins, antihypertensives and renin-angiotensin-aldosterone system (RAAS)-inhibitors, (II) potentially inappropriate drug prescribing, and (III) disease-specific medication burden with general HRQoL. Secondarily, the relationship with mental HRQoL will be explored.

## Materials and methods

A cross-sectional study was conducted using data from the e-VitaDM/ZODIAC study [17]. In short, 1,614 patients with T2D from 69 general practices in the Drenthe region of the Netherlands agreed to participate in a cohort study to investigate the effect of e-health on HRQoL. The database contains routinely collected data from the annual diabetes visits extracted from medical records from these patients. Furthermore, several questionnaires, including the EuroQol 5 Dimensions 3 Levels (EQ5D-3L) and the World Health Organization Well-Being Index (WHO-5) were filled out by the patients either at the general practice or at home in 2012. All patients with complete questionnaires, data registered during the yearly extensive diabetes control, and prescription data available were included in this study. All patients with a diagnosis date after 2012 were excluded from the analysis.

This study was approved by the Medical Ethical Review Committee of Isala, Zwolle, the Netherlands, and was registered under Clinictrials.gov number NCT01570140.

### Patient characteristics

The e-VitaDM/ZODIAC database includes structured data on age, gender, physical examination, laboratory measurements, diabetes-related complications and prescribed medication. Age and diabetes duration (categorized on recently diagnosed ≤ 2 years, less recently diagnosed 2–10 years, and older diagnosed > 10 years) were calculated using the date from the annual diabetes visit. Gender, body mass index (BMI) and smoking (smoking or non-smoking) were determined at the annual diabetes visit. Medication included glucose, blood pressure, and cholesterol lowering drugs. Comorbidities were grouped under coronary artery disease, including history of angina pectoris, coronary artery bypass grafting, myocardial infarct, percutaneous coronary intervention and heart failure, and cerebrovascular disease, including history of cerebrovascular accident and transient ischemic attack.

### Quality of prescribing

The quality of prescribing was assessed using the prescribing quality indicators which were systematically developed in the Netherlands. These quality indicators are based on the recommendations of the current clinical guidelines and have been validated for assessing the quality of prescribing in T2D patients. [3, 18] The set includes quality indicators focusing on appropriate start and intensification of treatment, current use of recommended treatment, use of preferred drugs and inappropriate prescribing.

#### Prescribing of recommended drugs

Sixteen indicators assessing the recommended prescribing of glucose lowering drugs, statins, antihypertensives and RAAS inhibitors were included [18]. These indicators focused on the start and intensification of glucose lowering drugs, statins, antihypertensives and RAAS inhibitors when recommended, on current prescribing of statins and RAAS inhibitors and preferred use of certain glucose lowering drugs and angiotensin-converting-enzyme inhibitors (ACE-i) (Table 1).

**Table 1:** Definition of the prescribing quality indicators divided into indicators focusing on recommended treatment and inappropriate prescribing and their included number of patients and outcomes.

Recommended prescribing		N included patients	Outcome
**1.**	The percentage of patients with T2D between 18 and 70 years with an elevated HbA_1c_ level (>53 mmol/mol) in the previous year, that started with glucose lowering drugs or reached the HbA_1c_ target level (≤53 mmol/mol)	10	80.0%
**2.**	The percentage of patients with T2D between 18 and 70 years treated with monotherapy metformin and with an elevated HbA_1c_ level (>53 mmol/mol) in the previous year, that is intensified with glucose lowering drugs or reached the HbA_1c_ target level (≤53 mmol/mol)	56	69.6%
**3.**	The percentage of patients with T2D between 18 and 70 years treated with two or more non-insulin glucose lowering drugs and an elevated HbA_1c_ level (>53 mmol/mol) in the previous year, that started with insulin or reached the HbA_1c_ target level (≤53 mmol/mol)	43	48.8%
**4.**	The percentage of patients with T2D 18 years or older that started with metformin among all starters of oral glucose lowering drugs	113	96.5%
**5.**	The percentage of patients with T2D 18 years or older treated with glucose lowering drugs that is prescribed metformin	846	92.1%
**6.**	The percentage of patients with T2D 18 years or older treated with two non-insulin glucose lowering drugs that is prescribed a combination of metformin and a SUD	287	86.1%
**7.**	The percentage of patients with T2D 18 years or older that started with gliclazide among all starters of SUD	47	19.2%
**8.**	The percentage of patients with T2D between 55 and 80 years old that is prescribed a statin	827	79.0%
**9.**	The percentage of patients with T2D between 18 and 80 years with an elevated LDL-cholesterol level (>2.5 mmol/l) in the previous year, that started with a statin or reached the LDL-cholesterol target level (≤2.5 mmol/l)	123	38.2%
**10.**	The percentage of patients with T2D between 18 and 80 years treated with simvastatin and with an elevated LDL-cholesterol level (>2.5 mmol/l) in the previous year, that switched to atorvastatin or rosuvastatin or reached the LDL-cholesterol target level (≤2.5 mmol/l)	101	49.5%
**11.**	The percentage of patients with T2D between 18 and 70 years with an elevated systolic blood pressure (>140 mmHg) in the previous year, that started with antihypertensives or reached the systolic blood pressure target level (≤140 mmHg)	43	74.4%
**12.**	The percentage of patients with T2D between 18 and 70 years treated with monotherapy antihypertensives and with an elevated systolic blood pressure (>140 mmHg) in the previous year, that is intensified with antihypertensives or reached the systolic blood pressure target level (≤140 mmHg)	59	62.7%
**13.**	The percentage of patients with T2D 18 years or older treated with two or more antihypertensives that is prescribed with an ACE-i or ARB	504	86.3%
**14.**	The percentage of patients with T2D between 18 and 70 years with micro- or macroalbuminuria in the previous year, that started with an ACE-i or ARB or returned to normo-albuminuria	12	50.0%
**15.**	The percentage of patients with T2D 18 years or older treated with antihypertensives and with micro- or macro-albuminuria that is prescribed an ACE-i or ARB	108	84.3%
**16.**	The percentage of patients with T2D 18 years or older that started with an ACE-i among all starters of RAAS treatment	105	64.8%
**Inappropriate prescribing**			
**17.**	The percentage of patients with T2D 18 years or older treated with SUD that is prescribed glibenclamide	311	1.0%
**18.**	The percentage of patients with T2D 18 years or older with an eGFR <30 ml/min/1.73m2 that is prescribed metformin	1	0.0%
**19.**	The percentage of patients with T2D 80 years or older with a normal HbA_1c_ level (<53 mmol/mol) that is prescribed two or more glucose lowering drugs	41	14.6%
**20.**	The percentage of patients with T2D 18 years or older treated with RAAS-inhibitors that is prescribed a combination of an ACE-i and ARB (dual RAAS blockade)	587	3.1%

T2D: type 2 diabetes; HbA_1c_: glycated hemoglobin; SUD: sulphonylurea derivative; LDL-cholesterol: low-density lipoprotein-cholesterol; ACE-i: angiotensin-converting-enzyme-inhibitor; ARB: angiotensin-receptor-blocker; RAAS: renin-angiotensin-aldosterone system; eGFR: estimated glomerular filtration rate

#### Prescribing of potentially inappropriate drugs

Four indicators assessing the prescribing of potentially inappropriate drugs were included [18]. They focused on prescribing of the non-recommended glibenclamide among sulfonylurea derivative users, prescribing of the contra-indicated metformin among patients with an impaired renal function (eGFR<30 ml/min/1.73m^2^), potential overprescribing of glucose-regulating drugs in elderly (≥ 80 years) with low HbA_1c_ values (HbA_1c_<53 mmol/mol), and prescribing of potentially unsafe dual RAAS blockade (Table 1).

### Disease-specific medication burden

To assess disease-specific medication burden, we calculated the burden of taking glucose, blood pressure, and cholesterol lowering drugs using a modified version of the Medication Regimen Complexity Index (MRCI) [19]. The MRCI comprises of three sections, which are giving burden scores for administration modality, dosing frequency and additional directions. For administration modality, the scores 1 and 4 were used for tablets and injections respectively. For dosing frequency, a score of 1 was used for drugs prescribed once daily, a score of 2 for drugs prescribed twice daily and so on. Furthermore, different scores are used for additional instructions for use. In our dataset, however, data on additional instructions (such as take with a specific fluid or at a specified time) was incomplete. Therefore we used a modified MRCI score, as has been proposed previously [20]. Data on the number of pills prescribed per time was complete and used in the analysis. A score of 1 was used for drugs which had multiple units per time or half a unit per time. Each prescribed drug received an overall score by adding the scores in the sections.

### Health-related quality of life

The primary outcome of this study was general HRQoL, assessed by the EQ5D-3L [21]. The EQ5D-3L consists of five questions regarding five dimensions: mobility, self-care, usual activities, pain/discomfort and anxiety/depression. For each question three answer categories are possible; no problems, some problems or extreme problems. This questionnaire has been validated for the Dutch population [22]. The outcome scores range from -0.333 to 1.0, where 1.0 represents perfect HRQoL. The secondary outcome of this study was mental HRQoL, assessed by the WHO-5 [23]. The WHO-5 consists of five questions regarding positive mood, vitality and general interest. For each question six answer categories are possible, ranging from constant to never. The WHO-5 score ranges from 0 to 100. Outcomes for both the EQ5D-3L and the WHO-5 questionnaires could only be calculated for completed surveys.

### Statistical analysis

Means with standard deviations are reported for normally distributed variables, medians with the inter-quartile range for non-normally distributed variables and percentages for categorical variables. Regression analysis was used to test for associations between the indicators of guideline-adherent prescribing and the HRQoL measures. The indicators are defined as binary variables, where 0 represents an included patient without a prescription for the treatment of interest and 1 represents a patient with a prescription. The residuals of the EQ5D-3L outcome were not normally distributed and therefore did not meet the assumption for linear regression. Since transformation of the variable did not improve the normality, we dichotomized this variable on the median EQ5D-3L score and logistic regression was performed for each indicator and the MRCI. The odds ratios with the 95% confidence intervals are reported for this analysis. The WHO-5 scores did satisfy the assumptions for performing linear regression. For this analysis, the effect sizes with 95% confidence intervals are reported. For both outcomes, two different models were assessed. Model 1 was the crude binary model including only the indicator of interest and the outcome. Model 2 tested whether the effects sizes of the associations were changed by possible confounders. The included confounders in all adjusted models were age, gender, diabetes duration, BMI, smoking status and history of coronary artery disease and cerebrovascular disease. None of these possible confounders are expected to lie in the causal pathway for the relationship of interest. Regression models for indicators including less than 50 patients were not assessed considering the low power, in particular for the adjusted models. P-values below 0.05 were considered statistically significant. All analyses were conducted using Stata version 14.2 Special Edition (Stata Corp., College Station, TX).

### Sensitivity analysis

A sensitivity analysis was performed where groups for HRQoL based on the EQ5D-3L were determined on a perfect score (= 1) compared to suboptimal score (<1).

## Results

Of the 1,614 patients that agreed to participate in the study, patients were excluded from the analysis because they did not have complete data on the EQ5D-3L or WHO-5 questionnaires (n = 423), there was no data available of the annual diabetes control visit (n = 125), and when there was no prescription data available (n = 22), leaving 1,044 primary care patients with T2D in this study. Of these, 1,035 completed the EQ5D-3L, and 1,011 the WHO-5 questionnaire. The patients were on average 65 years old, 44% was female and the median diabetes duration was 6 years. The mean HbA_1c_ was 50 mmol/mol, the average systolic blood pressure was 136 mmHg, the average LDL-cholesterol 2.4 mmol/l and the median albumin-creatinin ratio (ACR) was 0.7 mg/mmol. Furthermore, 82% of the patients were prescribed glucose lowering drugs, 75% blood pressure lowering drugs, and 78% statins. The score on the MRCI for these three therapeutic classes was on average 7.1 (standard deviation (SD): 4.1) (Table 2). The indicators focusing on start of glucose lowering drugs, intensification with insulin, preferred use of gliclazide among starters of SU-derivatives, start of antihypertensives, start of RAAS inhibitors, prescribing of metformin with impaired renal function and overprescribing of glucose-regulating drugs in the elderly included less than 50 patients and were therefore excluded from the further analysis (Table 1). The outcome of the indicators for recommended prescribing ranged from 38% to 97%, while the indicators on inappropriate prescribing ranged from 0% to 3%.

**Table 2:** Characteristics of the study population (2012).

Patient characteristics	Number of patients (%)	Mean ± standard deviation
Age (years)	1,044 (100)	65.2 (9.8)
≤55 years	152 (14.6)	49.0 (5.2)
55–80 years	827 (79.2)	66.7 (6.4)
>80 years	65 (6.2)	83.4 (2.6)
Female gender	458 (43.9)	
Diabetes duration (years)	1,036 (99.2)	6 [3; 10]*
≤ 2 years	254 (24.5)	1.0 (0.8)
2–10 years	586 (56.6)	6.4 (2.3)
>10 years (incl. missing values)	196 (18.9)	14.5 (4.9)
BMI (kg/m^2^)	1,031 (98.8)	29.9 (5.0)
Normal weight (≤25 kg/m^2^)	136 (13.0)	23.5 (1.2)
Overweight (25–30 kg/m^2^)	464 (44.4)	27.5 (1.4)
Obese (>30 kg/m^2^)	431 (41.3)	34.4 (4.3)
Smoking (yes)	156 (14.9)	
HbA_1c_ (mmol/mol)	1,037 (99.3)	49.6 (8.3)
LDL-cholesterol (mmol/l)	1,015 (97.2)	2.4 (0.8)
Systolic blood pressure (mmHg)	1,037 (99.3)	135.9 (15.2)
ACR (mg/mmol)	945 (90.5)	0.7 [0.3–1.5]*
eGFR (ml/min/1.73m^2^)	1,036 (99.2)	80.8 (12.1)
Poor kidney function (<30 ml/min/1.73m^2^)	1 (0.1)	28.7 (-)
Glucose-regulating drugs	853 (81.7)	
Metformin	785 (75.2)	
SU-derivatives	311 (29.8)	
Glibenclamide	3 (0.3)	
Insulin	141 (13.5)	
Blood pressure-regulating drugs	782 (74.9)	
Diuretics	346 (33.1)	
Beta-blockers	426 (40.8)	
Calcium channel blockers	179 (17.2)	
RAAS-inhibitors	587 (56.2)	
Statins	811 (77.7)	
Medication Regimen Complexity Index	1,044 (100)	7.1 (4.1)
Comorbidities		
CAD	203 (19.4)	
CBVD	71 (6.8)	
HRQoL questionnaires		
EQ5D-3L	1,035 (99.1)	0.86 [0.81–1.00]*
WHO-5	1,011 (96.8)	71.9 (17.8)

* Median with inter quartile range

ACR: albumin-creatinin ratio; BMI: body mass index; CAD: coronary artery disease; CBVD: cerebrovascular disease; EQ5D-3L: EuroQol 5 Dimensions 3 Levels; eGFR: estimated glomerular filtration rate; HRQoL: health-related quality of life; RAAS: renin-angiotensin-system; SU-derivatives: sulfonylurea derivatives; WHO-5: World Health Organization Well-Being Index

### EQ5D-3L

**Fig 1:**
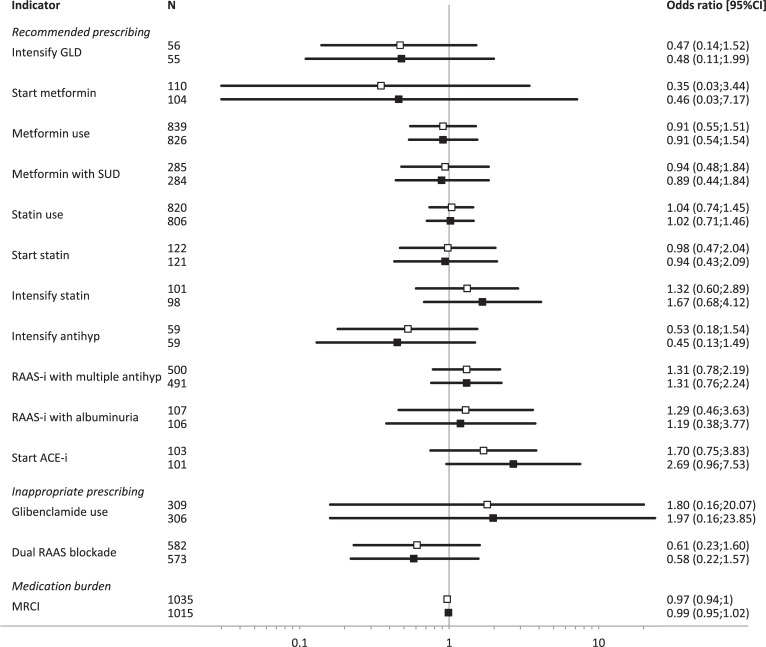
Overview of odds ratios of guideline-adherent prescribing quality indicators and medication burden with EQ5D-3L scores. □ represents unadjusted odds ratios; ∎ represents adjusted odds ratios; GLD: glucose lowering drugs; SUD: sulphonylurea derivatives; antihyp: antihypertensives; RAAS-i: rennin-angiotensin-aldosterone-system inhibitor; ACE-i: angiotensin-converting-enzyme-inhibitor; MRCI: medication regimen complexity index.

The median score of the total population on the EQ5D-3L questionnaire was 0.86 (interquartile range: 0.81–1.00) (Table 2). None of the indicators on recommended prescribing, or the indicators on inappropriate prescribing were significantly associated with EQ5D-3L scores in the logistic regression. Higher MRCI scores were significantly associated with lower EQ5D-3L scores. However, after adjustment for age, gender, diabetes duration, BMI and history of coronary disease this association lost significance (Fig 1). The sensitivity analysis using a perfect EQ5D-3L score versus all other scores showed similar results for all indicators expect for indicator 16 focusing on the preferred start with ACE-i among RAAS-i starters. A significant odds ratio of 3.09 (95% confidence interval: 1.11–8.59) was found for receiving this preferred treatment of ACE-i and a perfect score for the EQ5D-3L (S1 Fig).

### WHO-5

**Fig 2:**
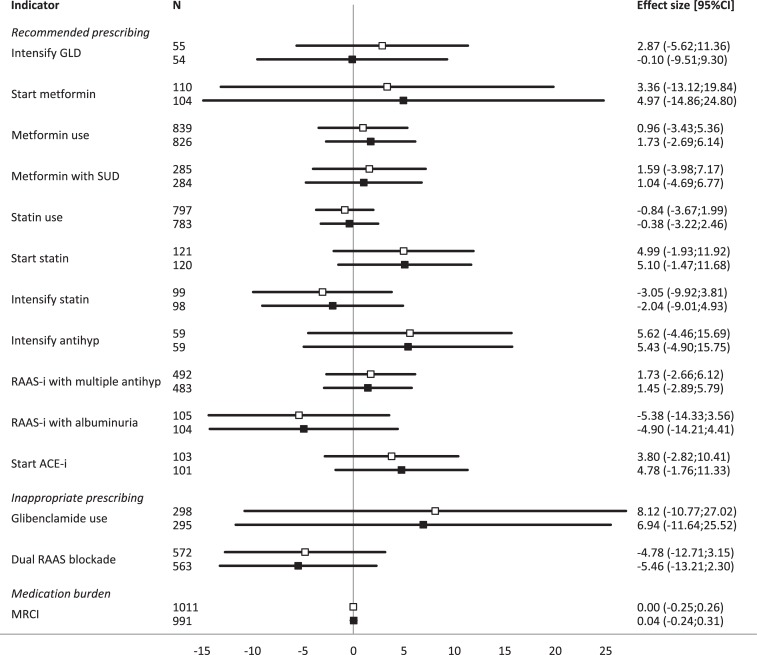
Overview of effect sizes of guideline-adherent prescribing quality indicators and medication burden with WHO-5 scores. □ represents unadjusted effect sizes; ∎ represents adjusted effect sizes; GLD: glucose lowering drugs; SUD: sulphonylurea derivatives; antihyp: antihypertensives; RAAS-i: rennin-angiotensin-aldosterone-system inhibitor; ACE-i: angiotensin-converting-enzyme-inhibitor; MRCI: medication regimen complexity index.

The mean score on the WHO-5 questionnaire of the total population was 72 (SD: 17) (Table 2). None of the indicators on recommended prescribing of RAAS-inhibitors or statins, or the indicators on inappropriate prescribing were significantly associated with higher or lower WHO-5 scores in linear regression. The MRCI for the glucose-, blood pressure-, and cholesterol-regulating drugs was also not associated with WHO-5 scores. Adjustments did not alter the results (Fig 2).

## Discussion

In this cross-sectional study among a selection of type 2 diabetes patients in The Netherlands, we found no evidence for associations between guideline-adherent prescribing of glucose lowering drugs, statins, antihypertensives and RAAS inhibitors and either general or mental HRQoL in T2D patients. Also prescribing of potentially inappropriate medication and having a higher disease-specific medication burden were not associated with HRQoL in patients with T2D.

Our study which uses prescribing quality indicators to assess recommended or inappropriate prescribing supports previous findings that prescribing more cardio-protective medication, as recommended by the guidelines, does not influence general or mental HRQoL [16]. This is in contrast to another study, where the association was observed between intensive multitherapy for cardiometabolic risk factors and better general HRQoL [15]. This multitherapy, however, included education and support for improving lifestyle, monthly visits and extensive blood glucose monitoring in addition to medication treatment. Therefore, it is unclear whether the medication treatment in itself influenced the HRQoL. Our findings suggest this may not be the case. The improved lifestyle and diabetes control might be responsible for the improved HRQoL, which has been shown before [24–27].

The prescribing of potentially inappropriate drugs in elderly patients has found to be associated with reduced general and mental HRQoL [10]. Such findings can be confounded by indication, that is, people who are prescribed more drugs, including potentially inappropriate drugs, may have a poorer health status, which in turn is associated with poorer HRQoL. Our study looked at the prescribing of specific inappropriate drugs in T2D patients, including glibenclamide and dual RAAS blockade. Our findings suggest that general or mental HRQoL is not affected by prescribing of such medication. This is, however, a cross-sectional study. Possibly, the patients receiving these potentially inappropriate drugs do not perceive any harm at that moment and therefore it did not affect their HRQoL. On the other hand, in this population, only three out of 311 identified patients at potential harm were prescribed glibenclamide, and only 18 out of 587 identified patients were prescribed dual RAAS blockade, which limited the power for these two analyses.

Surprisingly, we also did not find a significant association between the MRCI for glucose lowering drugs, statins and antihypertensives and general or mental HRQoL. Previously, a negative association was found between the overall MRCI and HRQoL in relatively young medication users [9]. Our finding suggests that in patients with a chronic disease, such as T2D, the disease-specific medication burden does not have a significant impact on their HRQoL.

We found no associations between guideline-adherent prescribing and HRQoL, at least when assessed with the EQ5D-3L and WHO-5. Furthermore, the direction of the non-significant effect estimates were not consistent when comparing the associations between the EQ5D-3L and the WHO-5. Previous research also used other questionnaires, such as the 36-item Short Form Survey. This makes it difficult to compare the results between studies and may explain the inconsistent results found previously. The EQ5D-3L is a widely used and accepted method to assess general HRQoL, and previously differences between treatments have been detected using the EQ5D-3L [12, 28]. The T2D patients in this study were relatively well controlled, which might influence the generalizability. On the other hand, the HRQoL was comparable to other T2D populations [14, 16].

This is a first study testing the association between quality indicators of guideline-adherent prescribing and HRQoL in T2D patients. This set of indicators has previously been validated for content, feasibility, and associations with intermediate outcomes [18]. An important strength as compared to other indicators to assess prescribing is that these indicators include only patients that have an indication for the treatment of interest and incorporate patient-specific information. Therefore, there is less bias of patients included in the indicators for whom the treatment of interest is not recommended or inappropriate.

Several limitations need to be addressed. First of all, due to the cross-sectional design it is not possible to assess cause-effect relationships between prescribing and HRQoL. We can thus only assess whether patients receiving different treatments at one point in time differed in HRQoL. We adjusted for several confounders, including diabetes duration and cardio- and cerebrovascular comorbidity, but we had limited data on other comorbidities that might have an influence on the patients’ HRQoL. Although such comorbidities in themselves are not necessarily associated with prescribing quality for diabetes, and therefore not confounders, the overall complexity of a patient can be a confounding factor in our study. It could reduce prescribing quality as measured by our indicators and also reduce HRQoL. By not adjusting for this factor we may thus overestimate the effects of prescribing quality on HRQoL. Another limitation is that the patients included in our analysis are a selection of all type 2 diabetes patients, since they were willing to participate in the e-VitaDM/ZODIAC study, had complete data and completed the questionnaires. One could speculate that these were younger and healthier patients but the average age and age distribution as well as their diabetes duration and risk factor control was as expected in the Dutch primary care population. In general, type 2 diabetes patients in The Netherlands were already well controlled in 2012 [29], which may lead to high quality of life scores. There was indeed little variation in the EQ5D-3L scores with a large proportion of the patients scoring high or perfect, which may have reduced the power to detect significant effects. Furthermore, because of a small number of patients included in several indicators these had to be excluded from the analyses. The indicators included in the analyses, however, covered a wide range of prescribing aspects.

In conclusion, we found no evidence that guideline-adherent prescribing and disease-specific medication burden are related to HRQoL in relatively well-controlled T2D patients. This gives no rise to refrain from prescribing guideline-recommended treatment in T2D patients, at least from a HRQoL perspective, but the interpretation of these results is limited by the cross-sectional study design and the selection of patients included in some indicators.

## Supporting information

S1 Fig Overview of sensitivity analysis calculating odds ratios of guideline-adherent prescribing quality indicators and medication burden with EQ5D-3L scores dichotomized on perfect and non-perfect scores. □ represents unadjusted odds ratios; ∎ represents adjusted odds ratios; GLD: glucose lowering drugs; SUD: sulphonylurea derivatives; antihyp: antihypertensives; RAAS-i: rennin-angiotensin-aldosterone-system inhibitor; ACE-i: angiotensin-converting-enzyme-inhibitor; MRCI: medication regimen complexity index.
(PDF)

S1 Dataset Supporting dataset including all necessary variables to perform the analyses from this manuscript. In this excel file, the first tab shows the actual dataset used for the analyses from this manuscript. The second tab describes all variables and their labels.
(XLSX)
