# Supplementary material for: Is guideline-adherent prescribing associated with quality of life in patients with type 2 diabetes?
Source: PLoS One. 2018 Aug 16;13(8):e0202319. doi: 10.1371/journal.pone.0202319 (PMC6095535; doi:10.1371/journal.pone.0202319)
Supplement: S1 Fig — □ represents unadjusted odds ratios; ∎ represents adjusted odds ratios; GLD: glucose lowering drugs; SUD: sulphonylurea derivatives; antihyp: antihypertensives; RAAS-i: rennin-angiotensin-aldosterone-system inhibitor; ACE-i: angiotensin-converting-enzyme-inhibitor; MRCI: medication regimen complexity index. (PDF) [file pone.0202319.s001.pdf]

S1 Fig: Overview of sensitivity analysis calculating odds ratios of guideline-adherent prescribing quality indicators and medication burden with EQ5D-3L scores dichotomized on perfect and non-perfect scores.

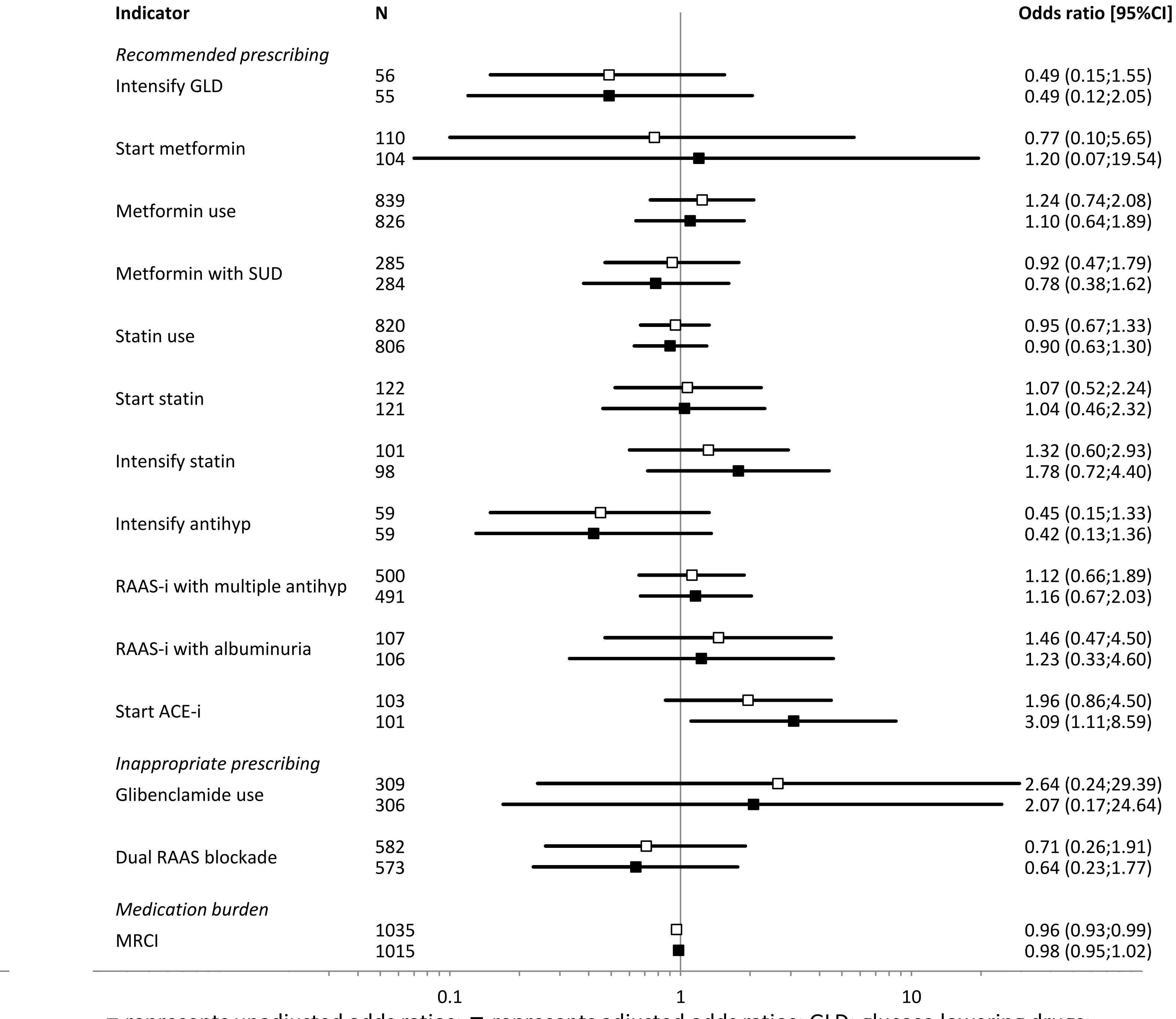

□ represents unadjusted odds ratios; ■ represents adjusted odds ratios; GLD: glucose lowering drugs; SUD: sulphonylurea derivatives; antihyp: antihypertensives; RAAS-i: rennin-angiotensin-aldosterone-system inhibitor; ACE-i: angiotensin-converting-enzyme-inhibitor; MRCI: medication regimen complexity index.
